# Beyond Half‐Cell Success: Cathode‐Electrolyte Reactivity Driving Magnesium Battery Full‐Cell Degradation at Elevated Temperature

**DOI:** 10.1002/advs.202511416

**Published:** 2025-08-04

**Authors:** Dedy Setiawan, Omar Falyouna, Toshihiko Mandai

**Affiliations:** ^1^ Research Center for Energy and Environmental Materials (GREEN) National Institute for Materials Science (NIMS) 1‐1 Namiki Tsukuba Ibaraki 305‐0044 Japan

**Keywords:** cathode, electrolyte, full‐cell, magnesium batteries

## Abstract

Rechargeable magnesium battery (RMB) is gaining attention as a promising alternative to lithium‐ion batteries, offering advantages such as low cost and high theoretical capacity of magnesium metal anodes. Yet, realizing stable, high‐voltage RMB full cells remains a considerable challenge. In this study, a full‐cell configuration is explored combining a vanadium oxide (VO_2_) cathode with a weakly coordinating anion‐based electrolyte. While encouraging performance is observed in half‐cell setups, translating it into full‐cell operation proves complex, particularly at elevated temperatures. At 60 °C, the initial discharge capacity of 77 mAh g^−1^ decreases notably to 28 mAh g^−1^ in the second cycle, whereas performance at 30 °C remains more stable ≈25 mAh g^−1^. Three‐electrode measurement suggests increasing overpotentials at the Mg anode as a key factor in the capacity degradation. Further analysis points to issues such as uneven Mg plating/stripping, surface pitting, and minor vanadium dissolution, contributing to impedance growth and cross‐over effects. These are linked to cathode–electrolyte side reactions, particularly under high‐voltage. Overall, the results emphasize the importance of developing stable interphases to enhance the long‐term performance of RMB full cells, especially at elevated temperatures.

## Graphical abstract

Despite several successful demonstrations in half‐cell tests reported in the literature, severe capacity degradation is observed in rechargeable magnesium battery (RMB) full cells employing oxide cathodes at elevated temperatures. Cathode–electrolyte reactivity leads to solvent‐derived electrolyte decomposition, unstable interphase formation, and transition metal dissolution, which in turn result in non‐uniform magnesium plating/stripping and increased overpotential.

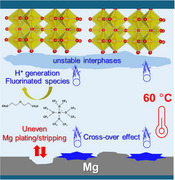

## 1 Introduction

Growing concerns over climate change and the increasing reliance on intermittent renewable energy sources such as solar and wind have intensified the global demand for reliable, high‐capacity electrochemical energy storage systems.^[^ ^1^, ^2^, ^3^, ^4^ ^]^ While lithium‐ion batteries (LIBs) have long been the preferred technology for mobile and stationary applications due to their high energy density, challenges including safety risks, high cost, and limited lithium availability have spurred the search for alternative energy storage solutions.^[^ ^5^, ^6^ ^]^ Consequently, research efforts have increasingly turned toward the development of next‐generation battery systems that incorporate more abundant, cost‐effective, and safer components, particularly those based on multivalent metal chemistries, as potential post‐LIB technologies.^[^ ^7^, ^8^, ^9^, ^10^ ^]^

Among these emerging alternatives, rechargeable magnesium battery (RMB) has attracted significant attention due to their potential for lower cost, enhanced safety, and high volumetric energy density when paired with high‐voltage cathodes.^[^ ^11^, ^12^, ^13^ ^]^ Magnesium (Mg) metal offers key advantages, such as lower cost, higher volumetric capacity, and a higher melting point than lithium.^[^ ^11^, ^14^ ^]^ Despite these merits, there is still a large gap in RMB research progress, particularly in realizing a high‐voltage RMB full cell. Although there has been considerable progress in the design of high‐voltage cathodes, compatible electrolyte systems, and Mg metal interface, most studies have focused primarily on half‐cell configurations.^[^ ^15^, ^16^, ^17^, ^18^, ^19^ ^]^ Meanwhile, to enable practical RMB, a reversible high‐voltage cathode must be paired with a Mg metal anode under lean electrolyte conditions. However, their compatibility and interfacial stability in full‐cell configurations remain poorly understood and require further study.

On the high‐voltage cathode side, several materials such as MgMn_2_O_4_, H_2_V_3_O_8_, VO_2_, and MgMnSiO_4_ have been extensively studied; however, they typically exhibit limited capacity at room temperature and often require elevated temperatures to activate Mg^2+^ intercalation due to kinetic constraints.^[^ ^20^, ^21^, ^22^, ^23^ ^]^ Nevertheless, their performance is often promising in half‐cells with non‐metallic counter electrodes and conventional electrolytes at elevated temperatures. In contrast, full‐cell studies with Mg metal anodes remain limited due to interfacial instability with conventional electrolytes.^[^ ^17^, ^18^, ^24^, ^25^ ^]^

On the electrolyte front, weakly coordinating anion systems (WCAs), such as those based on fluorinated alkoxyborate and alkoxyaluminate salts in ether‐based solvents, have shown promising compatibility with Mg metal.^[^ ^26^, ^27^, ^28^, ^29^, ^30^, ^31^, ^32^, ^33^, ^34^ ^]^ For example, a combination of Mg[Al(hfip)_4_]_2_ and diglyme exhibits stable cycling against Mg metal with very low overpotential (≈60 mV) and remarkable Coulombic efficiency over 99% after some activation pre‐cycle.^[^ ^34^ ^]^ This electrolyte system also has relatively high oxidation stability, over 3.5 V versus Mg/Mg^2+^, thus potentially compatible with high‐voltage cathodes.^[^ ^34^ ^]^ However, their performance in full‐cell configurations with such high‐voltage cathodes has not been widely evaluated.

In this study, we investigate the performance of an RMB full cell with the ethereal solutions of the representative WCAs as the electrolyte and VO_2_ as a cathode, at 30 and 60 °C. The goal is to bridge the gap between half‐cell investigations and full‐cell evaluation, as well as uncovering potential reactions on the cathode and the anode. Brookite‐type VO_2_ with a monoclinic‐type structure was selected as a model cathode due to its reversible Mg^2+^ intercalation capability and operating voltage, which lies within the electrochemical stability window of the chosen electrolyte.^[^ ^21^ ^]^ VO_2_ also exhibits limited capacity at room temperature and requires activation at elevated temperature, making it a suitable model for high‐voltage cathode with similar characteristics.^[^ ^21^ ^]^ Electrolyte solution of 0.3 m Mg[Al(hfip)_4_]_2_ in diglyme was chosen as the representative WCAs‐based electrolyte due to its remarkable Mg plating/stripping reversibility. The electrochemical performance of the electrolyte was first assessed at 30 and 60 °C, prior to full‐cell evaluation. Full cell evaluation was then conducted using VO_2_ as the cathode, 0.3 m Mg[Al(hfip)_4_]_2_ in diglyme as the electrolyte, and Mg metal as the anode.

## 2 Results

### 2.1 Electrolyte Performance at Elevated Temperature

**Figure 1:**
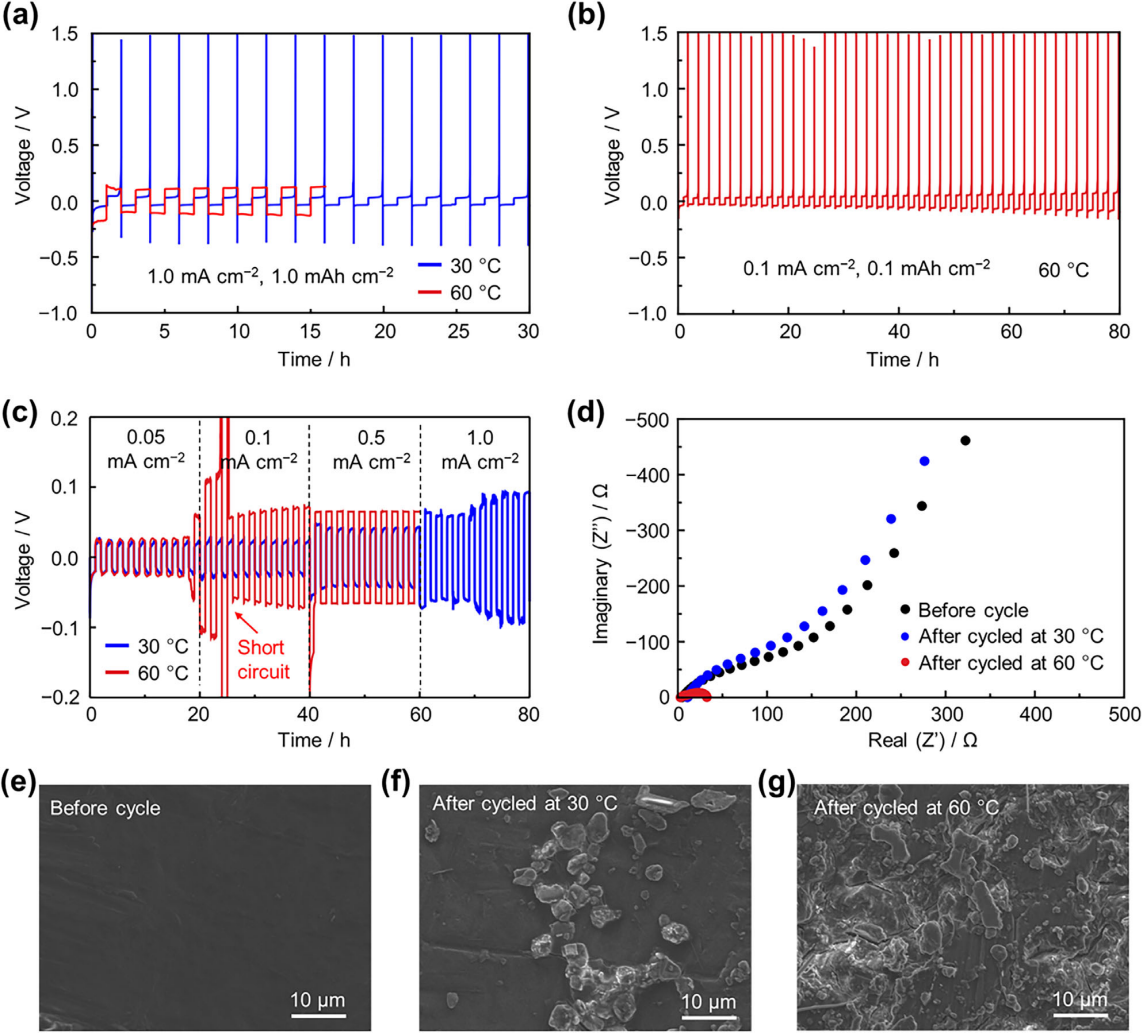
Galvanostatic discharge–charge profile of a) asymmetric cell test with Cu working electrode at 30 and 60 °C with current density of 1.0 mA cm^−2^, b) asymmetric cell test with Cu working electrode at 60 °C with current density of 0.1 mA cm^−2^, c) Mg metal symmetric cell with at various current densities at 30 and 60 °C. d) EIS profile of Mg metal symmetric cell before and after cycled at 30 and 60 °C. SEM images of Mg surface e) before cycle f) after cycled at 30 and g) after cycled at 60 °C.

The WCA‐based electrolyte system has been well developed, and reversible Mg plating/stripping behavior at room temperature has been widely reported. However, its performance at elevated temperatures remains largely unexplored. To investigate this, asymmetric cells were assembled using a Cu working electrode and Mg metal as both counter and reference electrodes, and tested at 30 and 60 °C to compare the Mg plating/stripping behavior. As shown in **Figure** **1**a and S2 (Supporting Information), typical Mg plating/stripping behavior was observed at 30 °C, with a Coulombic efficiency exceeding 96% at a current density of 1.0 mA cm^−2^ and a Mg utilization of 1.0 mAh cm^−2^, consistent with previous reports.^[^ ^34^ ^]^ In contrast, at 60 °C, the characteristic current spike during the stripping process was not observed, which suggests the occurrence of short‐circuiting phenomena.^[^ ^35^ ^]^

To further probe the electrochemical behavior at elevated temperature, asymmetric cells were tested at 60 °C under milder conditions (and 0.1 mA cm^−2^), as shown in Figure 1b and Figure S3 (Supporting Information). Under these conditions, the typical current spike during Mg stripping reappeared. However, the Coulombic efficiency was significantly lower, reaching only 78% in the first cycle and ≈90% in subsequent cycles (Figure S3, Supporting Information). This decrease in efficiency is likely due to enhanced electrolyte decomposition at the elevated temperature.

The Mg plating/stripping behavior in Mg metal symmetric cell was also evaluated. Figure 1c compares the overpotentials during Mg plating/stripping at various current densities. At a low current density of 0.05 mA cm^−2^, the overpotential remains ≈0.02 V at both temperatures and is stable over 20 cycles. However, when the current density is increased to 0.1 mA cm^−2^ after 20 cycles, the overpotential at 60 °C rises sharply to over 0.2 V, eventually leading to a short circuit.

Electrochemical impedance spectroscopy (EIS) results (Figure 1d) support this observation. After cycling at 60 °C, a very small semicircle appears, indicative of a short circuit.^[^ ^35^ ^]^ In contrast, only a slight increase in impedance is observed after cycling at 30 °C. The morphological changes on the Mg metal surface analyzed by scanning electron microscopy (SEM) after cycling are shown in Figure 1e (pristine), 1f (cycled at 30 °C), and 1 g (cycled at 60 °C). It has been previously reported that short circuits in Mg symmetric cells using WCAs‐based electrolytes are not caused by dendrite formation. Instead, they result from the non‐uniform 3D Mg deposits, which can penetrate the separator.^[^ ^36^, ^37^ ^]^ In this study, such non‐uniformity is clearly more pronounced after cycling at 60 °C. At higher temperatures, the rate of electrolyte decomposition increases, likely promoting the formation of unstable interphases.^[^ ^38^ ^]^ This leads to local overpotential variations and non‐uniform Mg plating.^[^ ^36^ ^]^ Additionally, the repeated inhomogeneous deposition and stripping cycles induce mechanical stress at the Mg‐electrolyte interface, potentially causing delamination or loss of interfacial contact. These combined effects explain the observed interfacial degradation and accelerated short circuit at higher current density. However, operating at lower current densities can mitigate these issues, though the stability of the interphases under such conditions still requires further investigation.

### 2.2 RMB Full Cell

**Figure 2:**
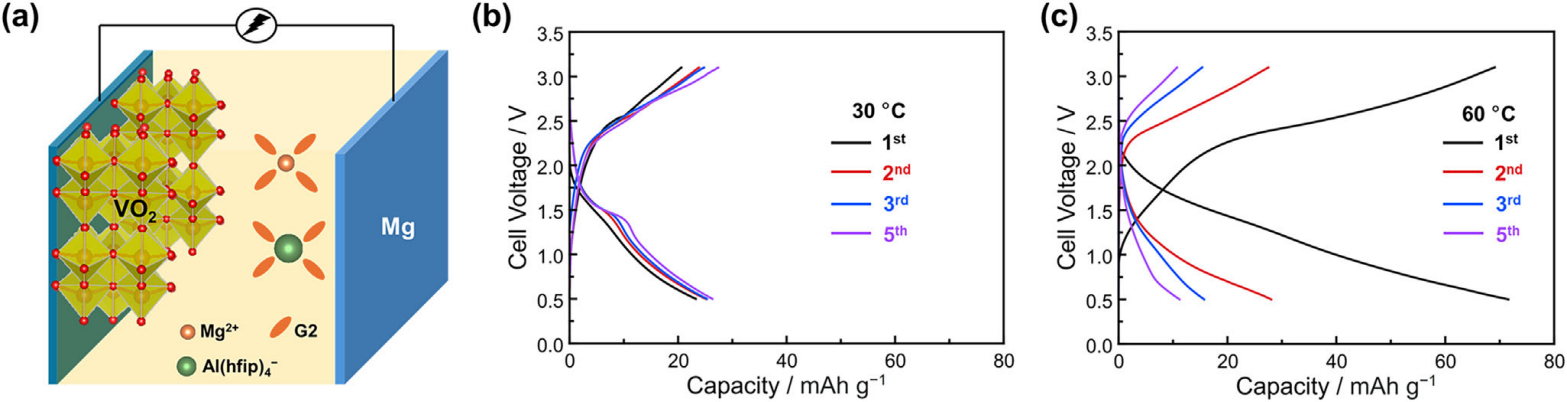
a) Illustration of RMB full cell with 0.3 m Mg[Al(hfip)_4_]_2_ in diglyme as the electrolyte and VO_2_ as the cathode. Galvanostatic discharge–charge profile of the full cell with a current density of 10 mA g^−1^ cycled at different temperatures; b) 30 °C and c) 60 °C.

A full cell was assembled using VO_2_ as the cathode, Mg metal as the anode, and 0.3 m Mg[Al(hfip)_4_]_2_ in diglyme as the electrolyte (**Figure** **2**a). Figure 2b presents the galvanostatic discharge/charge profile of the RMB full cell cycled at 30 °C with a current density of 10 mA g^−1^ based on the cathode active material, which corresponds to ≈0.01 mA cm^−2^ on the Mg metal anode. Under these conditions, the full cell delivers an initial discharge capacity of 23 mAh g^−1^ and a charge capacity of 20 mAh g^−1^. After five cycles, the capacity remains relatively stable at ≈26 mAh g^−1^. The limited capacity at 30 °C should correspond to the limited electrochemical activity of VO_2_, as reported previously.^[^ ^21^ ^]^

Figure 2c shows the corresponding discharge/charge profile at 60 °C under identical conditions with 30 °C. At this elevated temperature, the RMB full cell exhibits a significantly enhanced and reversible initial capacity of 77 mAh g^−1^. This improvement is likely due to more efficient Mg^2^⁺ diffusion within the VO_2_ structure at higher temperatures. However, this performance is not sustained over subsequent cycles. By the second cycle, the capacity drops sharply to 28 mAh g^−1^, followed by further declines to 16 mAh g^−1^ and 11 mAh g^−1^ in the third and fifth cycles, respectively. A similar degradation phenomenon after the 1st cycle was also observed in another WCAs‐based electrolyte, 0.3 m Mg[B(hfip)_4_]_2_ in diglyme, as shown in Figure S5 (Supporting Information). This pronounced capacity fading at elevated temperature highlights critical stability issues and motivates a deeper investigation into the degradation mechanisms in RMB full cells operating at 60 °C.

**Figure 3:**
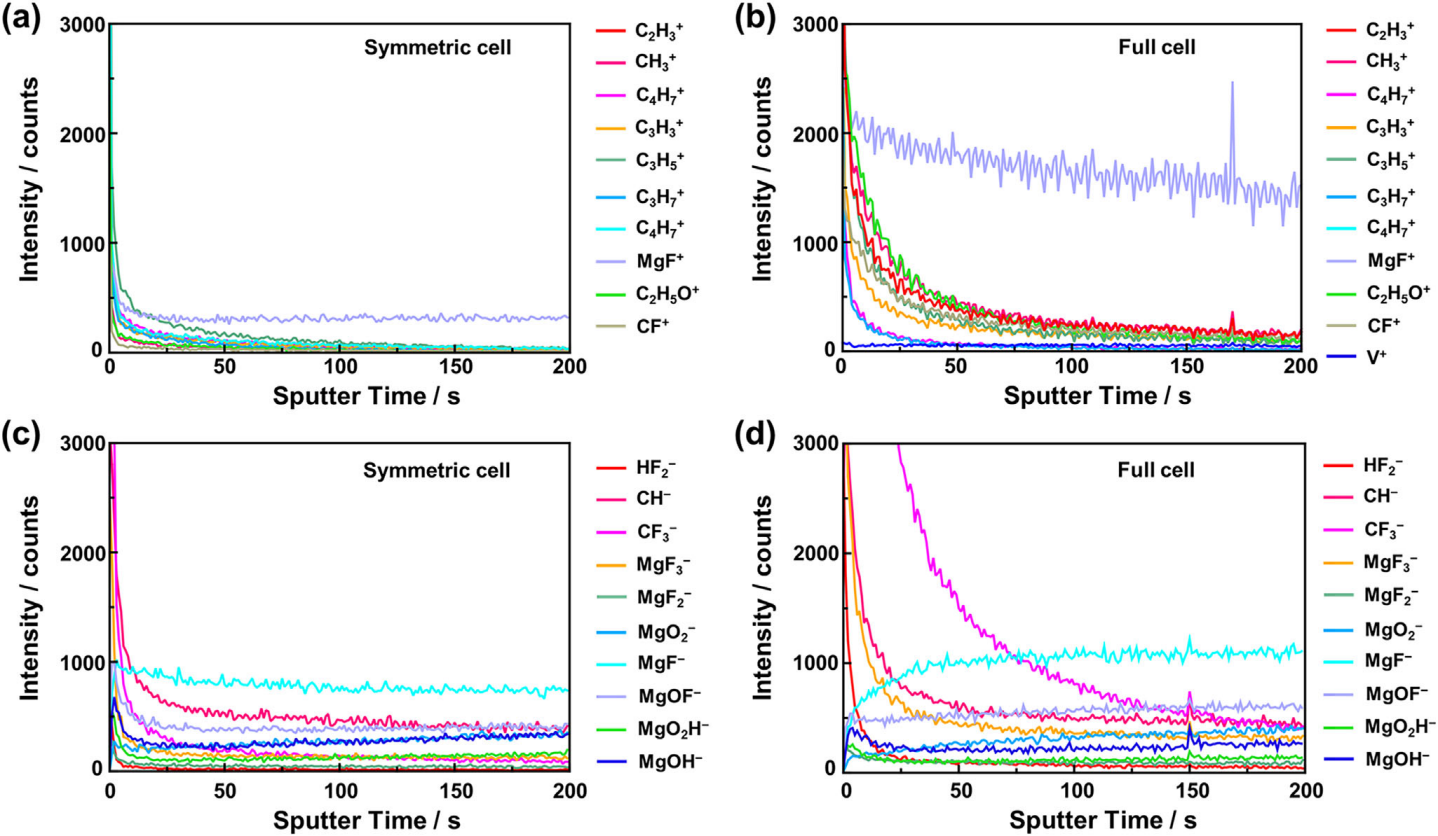
ToF‐SIMS analysis on Mg metal surface cycled with 0.3 m Mg[Al(hfip)_4_]_2_ in diglyme electrolyte in a), c) symmetric cell and b), d) in full cell with VO_2_ as a cathode, respectively.

We were initially curious about Mg metal surface composition cycled in a symmetric cell and in a full cell, because their performance might indicate a discrepancy. Therefore, time‐of‐flight secondary ion mass spectroscopy (ToF‐SIMS) analysis was performed on the Mg metal surface after cycling in both symmetric and full cells at 60 °C to investigate possible surface changes. **Figure** **3**a,c shows the ToF‐SIMS spectra of Mg metal cycled in the symmetric cell. Several organic‐based ions, MgOH^−^, MgO_2_H^−^, and fluorinated species were detected, suggesting solvent and anion‐derived electrolyte decomposition during cycling. Such decomposition is commonly observed in weakly coordinating anion‐type electrolytes.^[^ ^25^, ^39^, ^40^ ^]^ It is worth noting that the detected hydroxide‐related ions may also result from reactions with trace amounts of water present in the electrolyte, as previously reported.^[^ ^41^ ^]^

In contrast, the ToF‐SIMS spectra of Mg metal from the full cell (Figure 3b,d) reveals additional signals, including V⁺ ions, along with the decomposition products observed in the symmetric cell. Moreover, the intensities of organic‐based decomposition products as well as fluorinated species are significantly higher after full cell cycling, indicating a thicker and more complex interphase. These results highlight a clear difference in the interfacial chemistry of Mg metal when cycled in a symmetric cell versus a full cell at 60 °C, underscoring the critical impact of cathode‐electrolyte interactions in full cell configurations.

**Figure 4:**
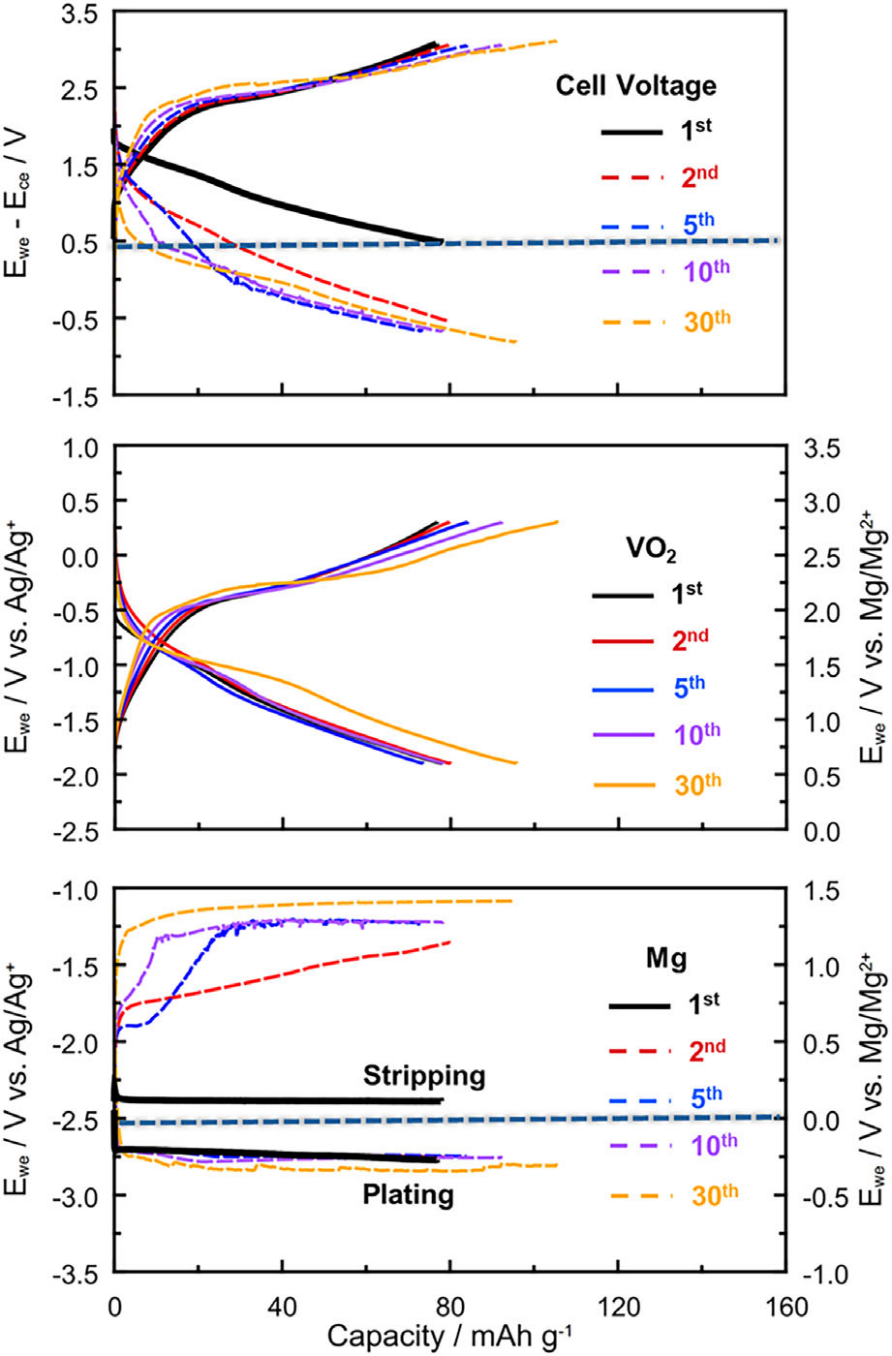
The three‐electrode cell measurement of VO_2_ as the working electrode, Mg metal as a counter electrode, and Ag/Ag^+^ as a reference electrode. The cycle was conducted with the current density of 10 mA g^−1^ at 60 °C. The horizontal line in the upper profile corresponds to the discharge cut‐off voltage of the corresponding full cell.

To investigate the observed discrepancies, we conducted a three‐electrode cell test to independently monitor the behaviors of the VO_2_ cathode and Mg metal anode during cycling at 60 °C. **Figure** **4** displays the galvanostatic discharge–charge profiles of the VO_2_ cathode, Mg anode, and the full cell. The first discharge capacity reached 77 mAh g^−1^, consistent with the full‐cell performance shown in Figure 2c. However, unlike the full cell, the capacity and discharge–charge overpotential of the VO_2_ cathode remained relatively stable in subsequent cycles. This result indicates good compatibility between the VO_2_ cathode and the 0.3 m Mg[Al(hfip)_4_]_2_ in diglyme electrolyte, but translating the performance into a full cell is non‐trivial.

In contrast, the Mg anode exhibited unstable behavior. While the initial overpotential was ≈0.3 V, it increased dramatically after the second cycle, exceeding 1 V, and continued to rise in later cycles. This instability suggests that undesirable reactions occur between the cathode and electrolyte during the initial cycle, adversely affecting the Mg plating/stripping process. One likely cause is the severe decomposition of the solvent at high‐voltage, which could trigger vanadium dissolution from the cathode and cause crossover effects, as observed in the ToF‐SIMS analysis earlier (Figure 3). We strongly believe that the rapid increase in Mg plating/stripping overpotential observed after the first cycle in the full cell is primarily due to the high‐voltage operation. As reported in a previous study using a WCA‐based electrolyte with a low‐voltage Mo_6_S_8_ cathode, interfacial degradation at the Mg anode was also detected, but the full cell remained operational for more than 20 cycles even at 120 °C.^[^ ^38^ ^]^ In that case, the interfacial failure was attributed mainly to the intrinsic thermal instability of the electrolyte and non‐homogeneous Mg plating/stripping rather than cathode‐driven degradation. Additionally, the rapid degradation of high‐voltage full cell after the 1st cycle due to transition metal cross‐over seems to be universally applicable to other high‐voltage cathodes.^[^ ^42^ ^]^ The transition metal dissolution at the cathode and subsequent migration to the Mg anode was also generally observed, not limited to only vanadium, but also in the case of cobalt.^[^ ^42^ ^]^ We hypothesize that solvent decomposition at high‐voltage raises the electrolyte's acidity, promoting transition metal dissolution, phenomena which are also well recognized in LIB systems.^[^ ^43^ ^]^ However, directly measuring the pH of the electrolyte during cycling remains challenging due to the lean electrolyte conditions used in this study and the lack of a suitable in situ experimental setup.

While transition metal dissolution typically leads to cathode capacity fading, the capacity of our VO_2_ cathode remained relatively stable. We believe this is due to the incomplete utilization of vanadium redox activity in the first cycle. Despite some vanadium loss, the remaining VO_2_ still retains redox‐active sites that become more accessible in later cycles. It is worth noting that the theoretical capacity of VO_2_, based on the V^3+^/V^4+^ redox couple, is 323 mAh g^−1^, whereas the observed first discharge capacity was only 77 mAh g^−1^. This suggests an activation process occurs during subsequent cycles, a behavior commonly reported for vanadium‐based cathodes.^[^ ^44^, ^45^ ^]^

On the Mg anode side, the parasitic reactions resulting from cathode–electrolyte reactivity led to non‐uniform plating/stripping, likely due to uneven current distribution across the Mg surface. Figure S6 (Supporting Information) shows the morphology of Mg metal after the first and tenth cycles: noticeable pitting and uneven Mg deposition appear after the first cycle and become more pronounced by the tenth. Additionally, SEM coupled with energy dispersive X‐ray spectroscopy(EDX)^−1^ mapping and ToF‐SIMS analysis on different spots after the first cycle (Figure S7, Supporting Information) confirm the presence of vanadium on the surface of the Mg metal anode.

### 2.3 Cathode Characterization

**Figure 5:**
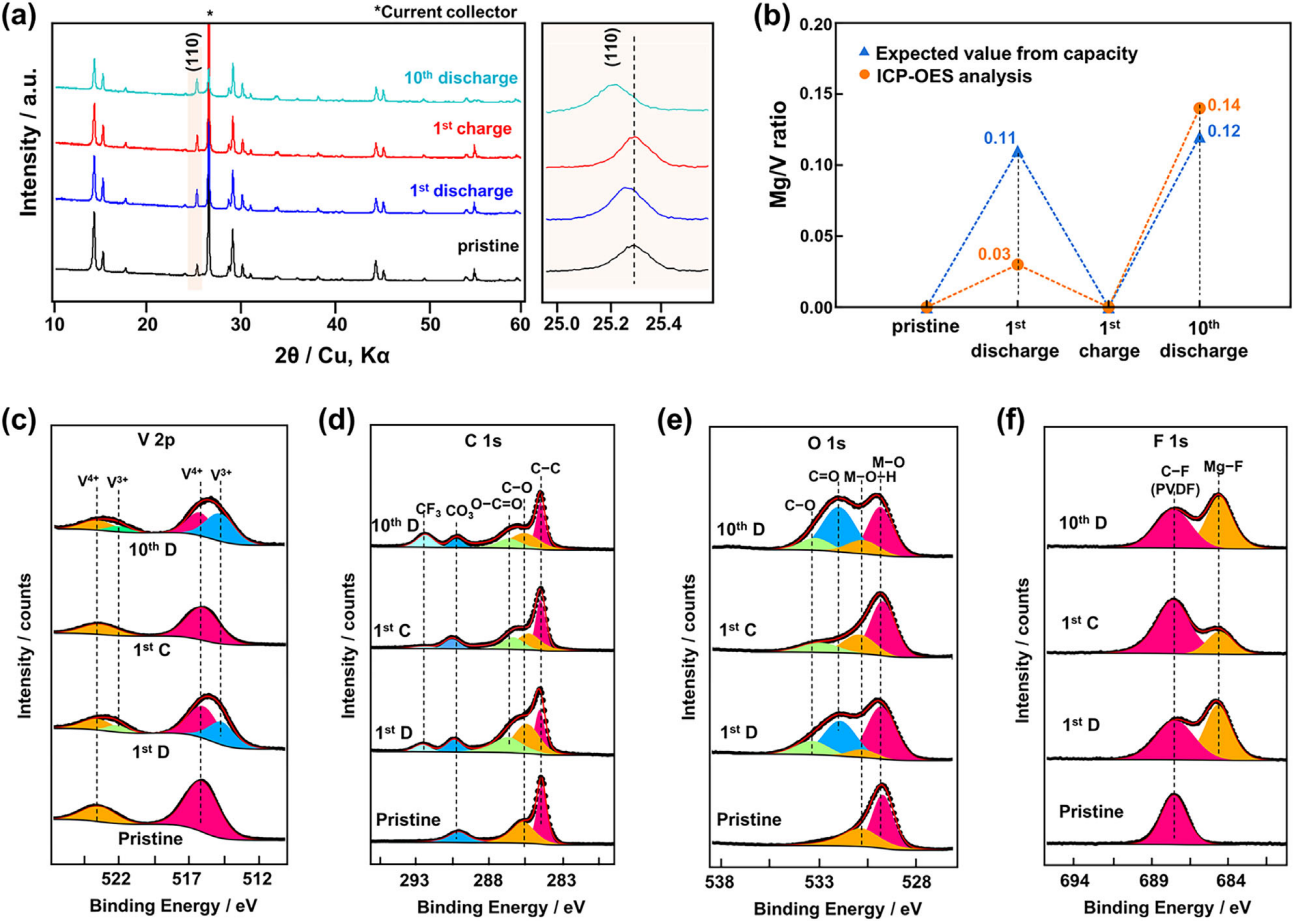
a) XRD profile of the VO_2_ cathode at pristine, 1st discharge, 1st charge, and 10th discharge with enlarged view of (110) peak. b) ICP‐OES analysis of the VO_2_ cathode at pristine, 1st discharge, 1st charge, and 10th discharge. Ex situ XPS of c) V 2p, d) C 1s, e) O 1s, and f) F 1s survey spectra of the VO_2_ cathode. 1st D, 1st C, and 10th D in the figures represent 1st discharge, 1st charge, and 10th discharge, respectively.

To better understand the cathode–electrolyte reactivity in the full cell, particularly during the first cycle, which appears to trigger vanadium dissolution, we conducted structural and elemental analyses on the VO_2_ cathode. **Figure** **5**a shows X‐ray diffraction (XRD) profiles of the VO_2_ cathode at four stages: pristine, after the first discharge, first charge, and tenth discharge. Following the first discharge, the (110) diffraction peak shifts slightly to a lower angle, consistent with previous reports, indicating ion insertion into the VO_2_ structure. This peak returns to its original position after the first charge, suggesting a reversible process. Interestingly, after the tenth discharge, the (110) peak shift is more pronounced than after the first discharge, despite their comparable capacities. This suggests that more ions were inserted into VO_2_ by the tenth discharge.

To quantify the extent of Mg^2^⁺ insertion, inductively coupled plasma optical emission spectroscopy (ICP‐OES) analysis was performed. Figure 5b presents the measured Mg/V ratios at the same four stages, compared with expected values derived from galvanostatic discharge–charge capacities in the three‐electrode setup. After the first discharge, the measured Mg/V ratio is only 0.03, significantly lower than the expected 0.11, implying that most of the initial capacity is not due to Mg^2^⁺ intercalation. A plausible explanation is the contribution of H⁺ insertion and/or the formation of cathode–electrolyte interphases. Despite the use of a low water content electrolyte, electrochemical decomposition of the ether‐based solvent may generate trace amounts of H_2_O, facilitating H^+^ formation and interphase growth.^[^ ^40^, ^46^ ^]^ On the other hand, the Mg/V ratio after the tenth discharge is 0.14, which is consistent with the expected value of 0.12, supporting the XRD observation of greater ion insertion in the later cycle.

It is worth noting that the high Coulombic efficiency in the first cycle appears contradictory to the occurrence of side reactions during discharge. However, electrolyte decomposition—and hence side reactions—may occur not only during discharge but also during charge, since the cathode‐electrolyte interphases are not stable enough to prevent the electron transfer at the cathode interface. Although the Coulombic efficiency appears high, it can be inferred that side reactions during the initial charge process contributed a comparable amount of electron transfer as the discharge process in the first cycle. This gives the false impression that the charge and discharge capacities arise from a reversible reaction within the applied cut‐off voltage, when in fact they do not. This interpretation is supported by the consistent observation that the charge capacity exceeds the discharge capacity in subsequent cycles (Figure 4). Therefore, a portion of the electron transfer during the first charge originates from irreversible side reactions rather than a reversible process.

To further probe the redox mechanism and the cathode–electrolyte interphase (CEI), ex situ X‐ray photoelectron spectroscopy (XPS) was carried out on the VO_2_ cathode after the first discharge, first charge, and tenth discharge. As shown in the V 2p spectra (Figure 5c), both V^3+^ and V^4+^ signals are present after the first and tenth discharges, even though only a small amount of Mg was inserted in the first cycle. This suggests that vanadium reduction may be partially driven by other species, such as protons. The C 1s, O 1s, and F 1s spectra (Figure 5d–f) reveal various organic and inorganic decomposition products on the cathode surface after the first discharge, corroborating the conclusion from XRD and ICP‐OES that much of the initial capacity arises from side reactions. After the first charge, some of these decomposition products, particularly fluorinated species, appear to diminish, likely due to further decomposition at high‐voltage and increased electrolyte acidity, which may dissolve part of the CEI. The dissolution of CEI components and vanadium into the electrolyte after the first charge could be a key factor contributing to the increased overpotential observed at the Mg anode. These dissolved species may migrate to the Mg anode, disrupting uniform Mg plating/stripping and increasing interfacial resistance. However, a fraction of the CEI remains intact, which may help suppress side reactions in subsequent cycles and promote more effective Mg^2^⁺ intercalation during discharge.

### 2.4 The Impact of Cut‐Off Voltage

**Figure 6:**
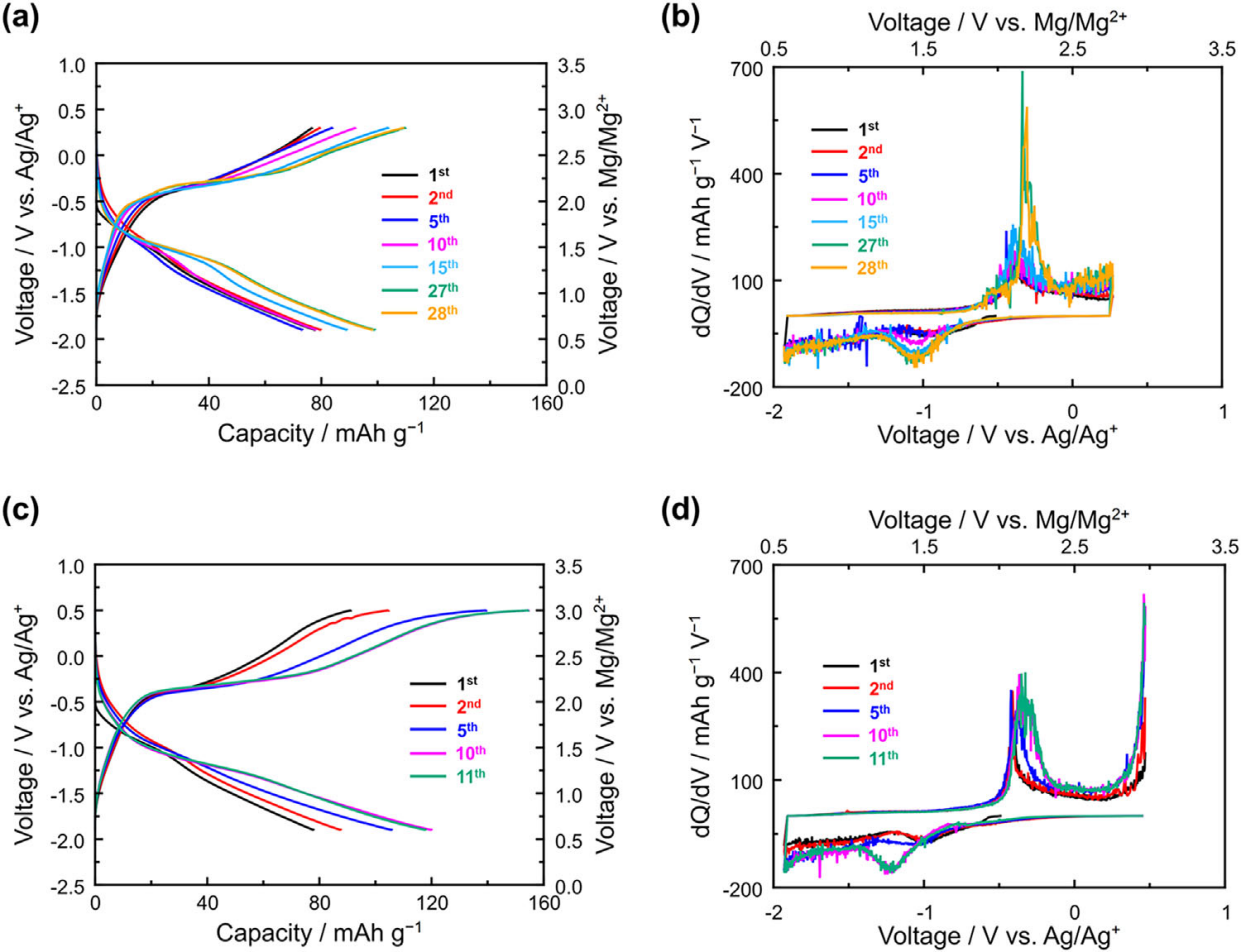
a) Galvanostatic discharge–charge of VO_2_ working electrode at three‐electrode cell test with a current density of 10 mA g^−1^ cycled with different charge cut‐off voltage of (a) 0.3 V versus Ag/Ag^+^ and c) 0.5 V versus Ag/Ag^+^. The corresponding dQ/dV of the discharge–charge profile are shown in b) for 0.3 V versus Ag/Ag^+^ and d) for 0.5 V versus Ag/Ag^+^.

We were further interested in the cathode–electrolyte reactivity during the charging process, as the dissolution of some CEI components tends to occur at this stage. To investigate this, we studied the impact of the charge cut‐off voltage by intentionally increasing it to a higher value. **Figure** **6**a shows the galvanostatic discharge–charge profile of VO_2_ with a charge cut‐off voltage of 0.3 V versus Ag/Ag⁺ (2.79 V vs Mg/Mg^2+^), while the corresponding dQ/dV curve is presented in Figure 6b. At this cut‐off voltage, the discharge–charge capacity remains relatively stable during the initial cycles. The capacity gradually increases to 90 mAh g^−1^ by the 27^th^ cycle and then saturates. The dQ/dV plot shows no indication of anodic decomposition of the electrolyte up to 0.3 V versus Ag/Ag⁺.

In contrast, when the charge cut‐off voltage was increased to 0.5 V versus Ag/Ag⁺ (2.99 V vs Mg/Mg^2+^), the cell exhibited a higher charge capacity (91 mAh g^−1^) than discharge capacity (77 mAh g^−1^) in the first cycle. In subsequent cycles, the discharge capacity rapidly increased, reaching 120 mAh g^−1^ after 10 cycles, with charge capacities consistently higher than discharge capacities. This suggests the presence of side reactions contributing to the excess charge capacity at 0.5 V. The dQ/dV profile in Figure 6d supports this hypothesis, showing a noticeable current spike above 0.3 V versus Ag/Ag⁺. These results indicate that even within the nominal electrochemical stability window of the electrolyte, side reactions at the cathode become more pronounced after reversible cycling is established.

**Figure 7:**
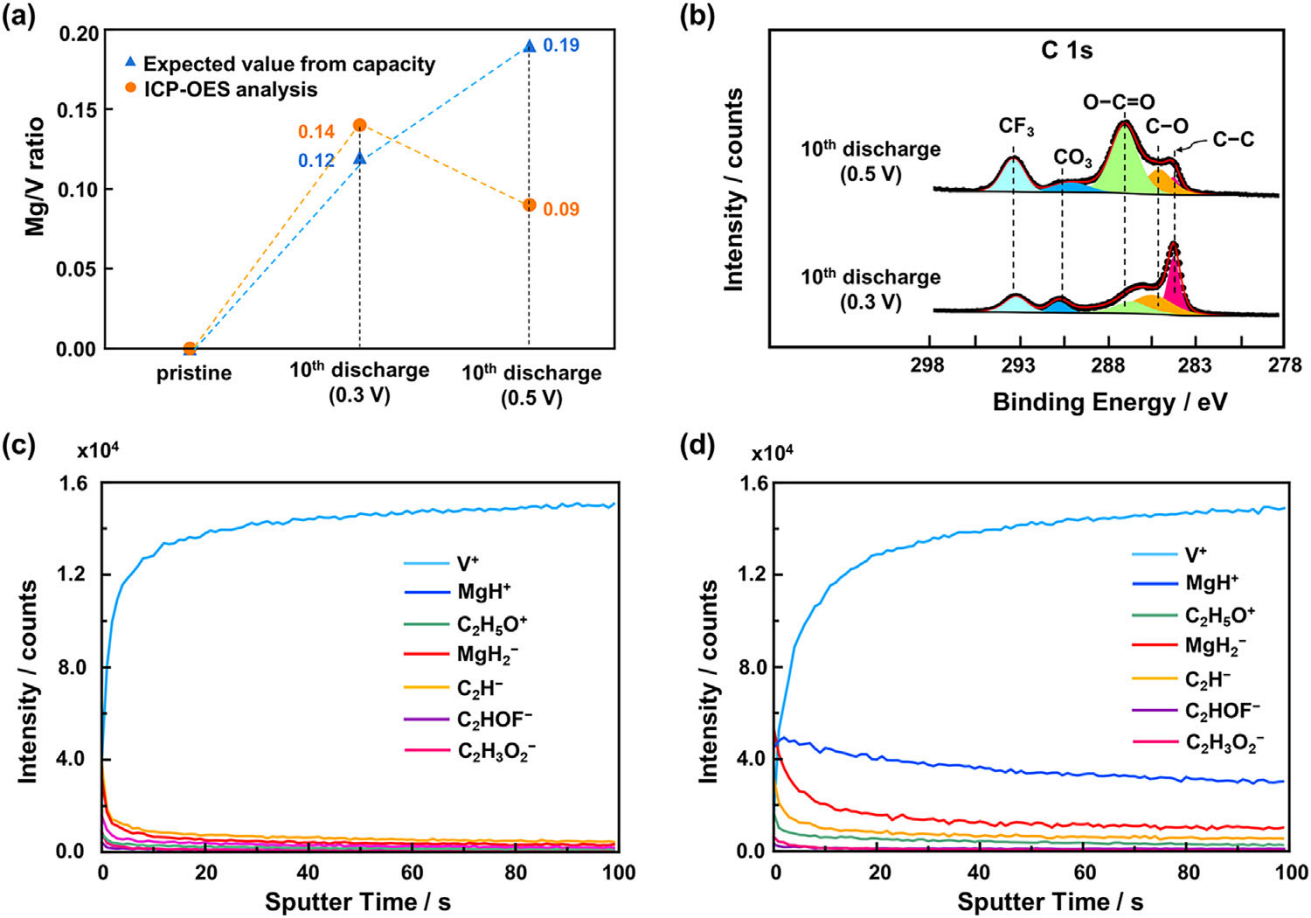
a) ICP‐OES analysis and b) XPS C1s survey spectra of VO_2_ at 10th discharge after cycled with the charge cut‐off voltage of 0.3 and 0.5 V versus Ag/Ag⁺. ToF‐SIMS analysis of VO_2_ surface cycled with the charge cut‐off voltage of c) 0.3 V and d) 0.5 V versus Ag/Ag⁺.

To further explore the effects of the increased cut‐off voltage, various elemental analyses were conducted. ICP‐OES results (**Figure** **7**a) show that Mg insertion after the 10th discharge at a 0.5 V versus Ag/Ag⁺ cut‐off is only 0.09, significantly lower than the expected value of 0.19. This confirms that, unlike at 0.3 V, continuous side reactions occur during cycling when the cut‐off voltage exceeds the VO_2_ reversible capacity.

XPS analysis of the C 1s spectra (Figure 7b) revealed a greater accumulation of both organic and inorganic CEI species on the VO_2_ cathode cycled at 0.5 V, particularly O─C═O and CF_3_ moieties. ToF‐SIMS analysis (Figure 7c,d) showed similar types of CEI species for both voltage conditions, but with notably higher intensity after cycling at 0.5 V. A strong signal corresponding to MgH_2_^−^ was also observed, likely originating from H⁺ intercalation during discharge. These CEI components predominantly accumulate on the surface of VO_2_ and may readily dissolve into the electrolyte, subsequently migrating to the Mg anode and contributing to interfacial instability.

## 3 Conclusion

We have demonstrated RMB full cell employing 0.3 m Mg[Al(hfip)_4_]_2_ in diglyme as an electrolyte and VO_2_ as a model cathode, tested at both 30 and 60 °C. Our study reveals that cathode–electrolyte reactivity is a key factor contributing to cell degradation at elevated temperature (60 °C). During discharge, side reactions occur concurrently with Mg^2+^ intercalation, leading to the formation of organic and inorganic CEI. During the subsequent charge process, parts of the CEI and vanadium species dissolve from the cathode and migrate to the Mg anode, significantly increasing the Mg plating/stripping overpotential in later cycles. Our finding indicates that the electrochemical stability window of the electrolyte itself does not guarantee a stable cycling of high‐voltage cathode under full cell operation, especially at elevated temperature.

Moreover, we found that the choice of charge cut‐off voltage strongly influences the extent of these cathode‐electrolyte side reactions, affecting both discharge and charge performance of the full cell. These findings highlight a critical challenge in translating half‐cell performance to full‐cell systems in RMB development particularly under elevated temperature. Future research should focus on improving the electrochemical stability of electrolytes to advance practical RMB applications.

## 4 Experimental Section

### Electrolyte and Cathode Preparation

0.3 m Mg[Al(hfip)_4_]_2_ in diglyme electrolyte was prepared by dissolving Mg[Al(hfip)_4_]_2_ salt into diglyme solvent and stir at room temperature inside an Ar‐filled glovebox. Mg[Al(hfip)_4_]_2_ salt preparation was reported elsewhere.^[^ ^34^ ^]^ Diglyme (>99.5%, Kanto Chemical CO., INC., Japan) was treated with molecular sieves for overnight prior to electrolyte preparation. The water content of the electrolyte was less than 50 ppm, as measured by Karl‐Fischer moisture titration (MKC710, KEM).

VO_2_ powder was prepared following the previous method.^[^ ^21^ ^]^ 0.3638 g of V_2_O_5_ (>99.0%, Kanto Chemical CO., INC., Japan) was mixed with 0.432 g of oxalic acid (≥99.0%, Sigma–Aldrich) in 50 mL of D.I. water at 40 °C for 24 h. After 24 h of stirring, the color of the solution transformed from light green to dark blue. The solution was subjected to hydrothermal reaction in a 100 mL Teflon‐lined stainless‐steel autoclave and heated at 180 °C for 24 h. The resulting precipitates were filtered, washed with D.I. water, and dried at 80 °C for 6 h under vacuum conditions. The purity of VO_2_ was confirmed using powder XRD (SmartLab, Rigaku, Japan) with Cu Kα X‐ray tube, and refined using the powder X‐ray Rietveld refinement program GSAS, and shown in Figure S1a (Supporting Information).^[^ ^47^ ^]^ The morphology of VO_2_ was also confirmed using SEM (SU8200, Hitachi, Japan), and shown in Figure S1b (Supporting Information).

Cathode material was prepared by mixing VO_2_ powder, Acetylene Black, and PVDF binder with a mass ratio of 8: 1: 1, dispersed in *N*‐methyl‐2‐pyrrolidone (NMP), and casted on to carbon‐coated aluminum foil as a current collector. The average loading mass of the cathode was 1 mg cm^−2^.

### Electrochemical Characterization

Cathode active material (2.01 cm^2^), a glass fiber separator (GF/D, Whatman) (2.01 cm^2^), and polished Mg metal (2.01 cm^2^) were assembled in a two‐electrode cell for full cell test, and a three‐electrode cell for half‐cell test. In the three‐electrode cell test, Ag/Ag^+^ reference electrode was used, and calibrated as 2.49 V versus Mg/Mg^2+^.^[^ ^34^ ^]^ The amount of electrolyte for each cell was 200 µl.

The full cell discharge–charge measurement was conducted using a battery cycler (HJ1001SD8 C, HD Meiden Hokuto, Japan). While EIS and the three‐electrode cell measurement were conducted using EC‐Lab software on a Biologic VMP3 multichannel potentiostat (Biologic Science Instruments SAS). The cut‐off voltage of the full cell was 0.5 to 3.1 V, and the three‐electrode cell was −1.9 to 0.3 V versus Ag/Ag^+^, which are still in the range of the electrochemical stability window of the electrolyte (Figure S4, Supporting Information).

### Cathode and Anode Characterization

The surface of the Mg anode and VO_2_ cathode after cycles was analyzed using SEM (SU8200, Hitachi, Japan) equipped with EDX, XPS (VersaProbe II, ULVAC‐PHI), and ToF‐SIMS (ToF‐SIMS5‐AD‐GCIB) with a primary ion gun of 30 kV Bi^3++^ and 4 nm min^−1^ as SiO_2_ film sputter rate. The electrochemical cells were disassembled inside an Ar‐filled glovebox. The electrodes were washed with dimethoxyethane (DME) and dried for several hours inside the glovebox. All interfacial characterizations, including the sample transfer process, were conducted under an air‐ and moisture‐free atmosphere.

## Conflict of Interest

The authors declare no conflict of interest.

## Supporting information



Supporting Information

## Data Availability

Data sharing is not applicable to this article as no new data were created or analyzed in this study.
