# Supplementary material for: Beyond Half‐Cell Success: Cathode‐Electrolyte Reactivity Driving Magnesium Battery Full‐Cell Degradation at Elevated Temperature
Source: Adv Sci (Weinh). 2025 Aug 4;12(40):e11416. doi: 10.1002/advs.202511416 (PMC12561278; doi:10.1002/advs.202511416)
Supplement: Supplementary file 1 — Supporting Information [file ADVS-12-e11416-s001.pdf]

Supporting Information

Beyond Half-Cell Success: Cathode-Electrolyte Reactivity Driving Magnesium Battery Full-Cell Degradation at Elevated Temperature

Dedy Setiawan, [A] Omar Falyouna [A] and Toshihiko Mandai*, [A]*

A. Research Center for Energy and Environmental Materials (GREEN), National Institute for Materials Science (NIMS), 1-1 Namiki, Tsukuba, Ibaraki, 305-0044 Japan

E-mail: SETIAWAN.Dedy@nims.go.jp, MANDAI.Toshihiko@nims.go.jp

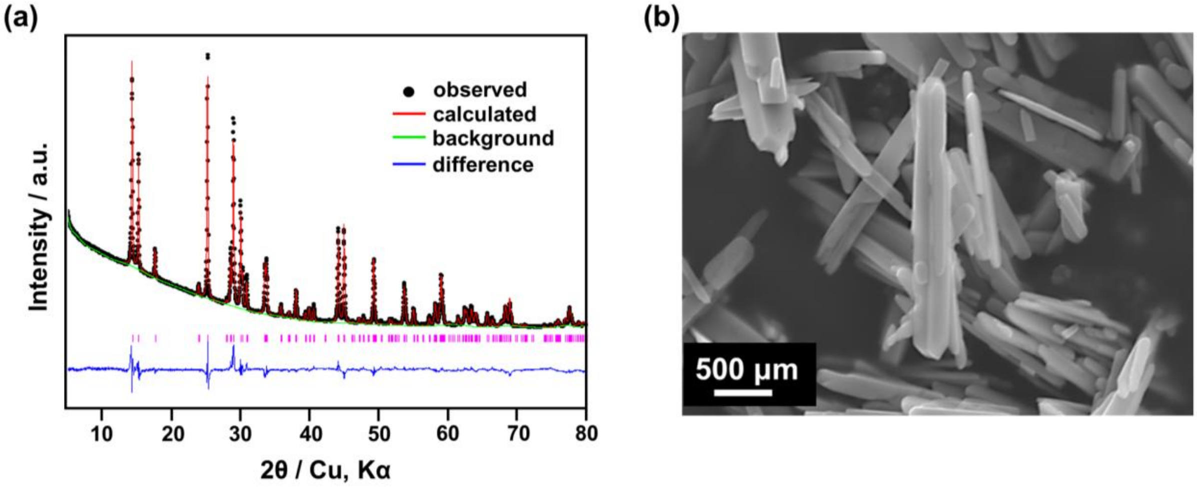

Figure S1. (a) X-ray Rietveld refinement of VO_2 powder. Space group : C 2/m, a = 12.065 (1) Å, b = 3.690 (1) Å, c = 6.419 (5) Å, β = 106.99 (1)°. (b) SEM image of VO_2 powder.

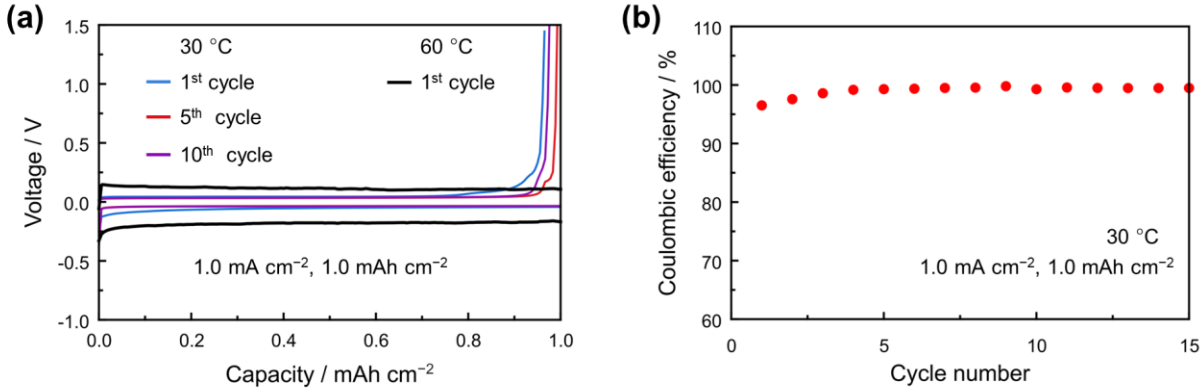

Figure S2. Asymmetric cell test comparison employing Cu as a working electrode and Mg metal as counter and reference electrode at 30 °C and 60 °C with a current density of 1.0 mA cm^{-2}. (b) The corresponding Coulombic efficiency at 30 °C.

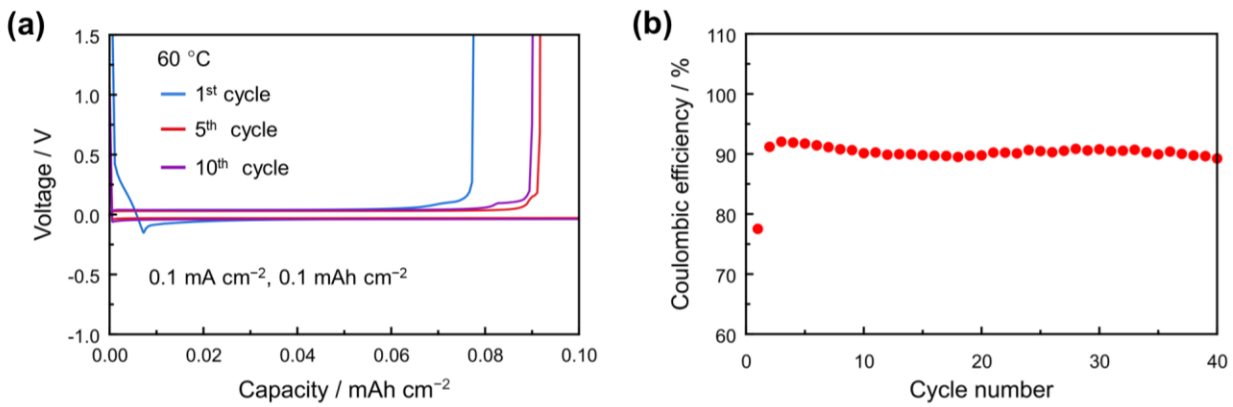

Figure S3. (a) Asymmetric cell test employing Cu as a working electrode and Mg metal as counter and reference electrode at 60 °C with a current density of 0.1 mA cm^{-2}. (b) The corresponding Coulombic efficiency of the asymmetric cell test.

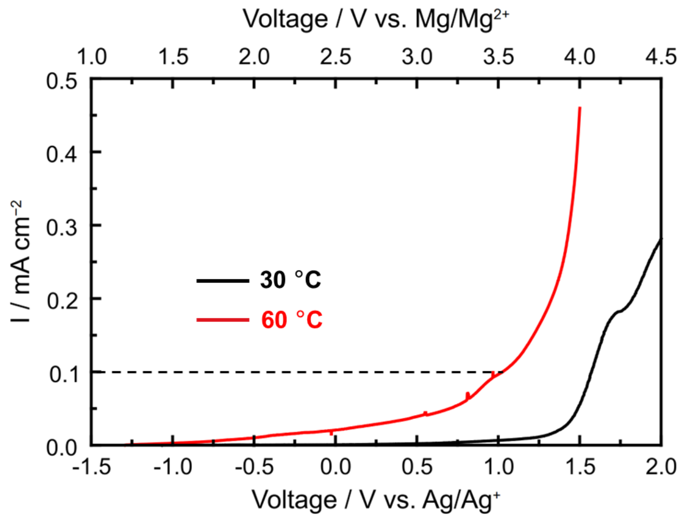

Figure S4. LSV profile of 0.3 M $Mg[Al(hfip)_4]_2$ in diglyme with carbon-coated Al foil as working electrode.

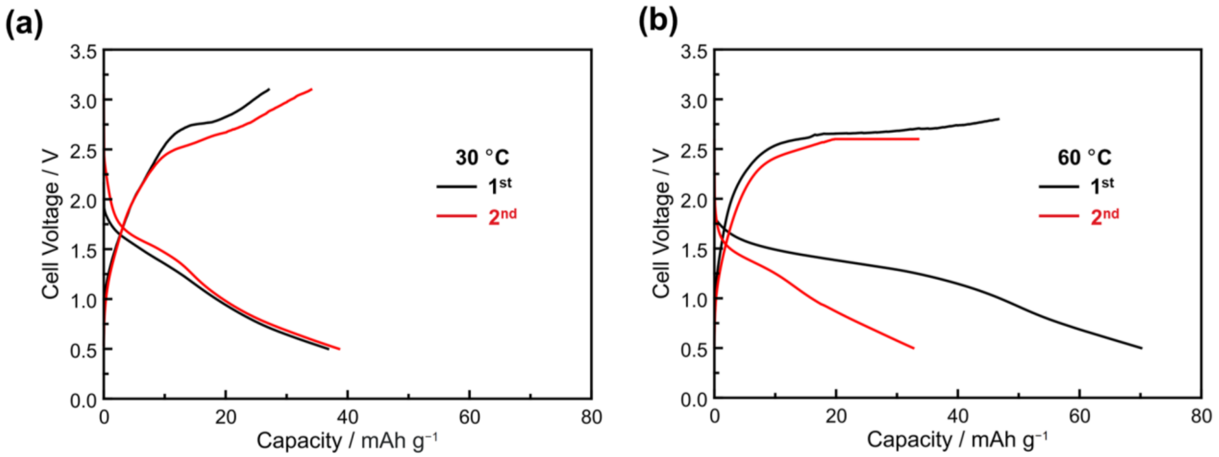

Figure S5. Galvanostatic discharge-charge profile of the full cell comprising VO_2 cathode and 0.3 M $Mg[B(hfip)_4]_2$ in diglyme as the electrolyte with a current density of 10 mA g^{-1} cycled at different temperatures; (a) 30 °C and (b) 60 °C.

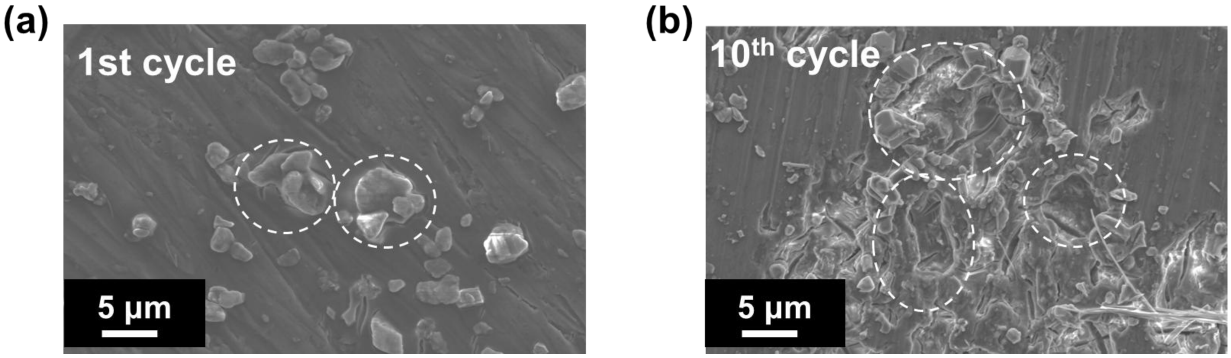

Figure S6. SEM image of Mg metal anode surface cycled in three-electrode cell with VO_2 as the cathode (a) after 1st cycle and (b) after 10th cycle.

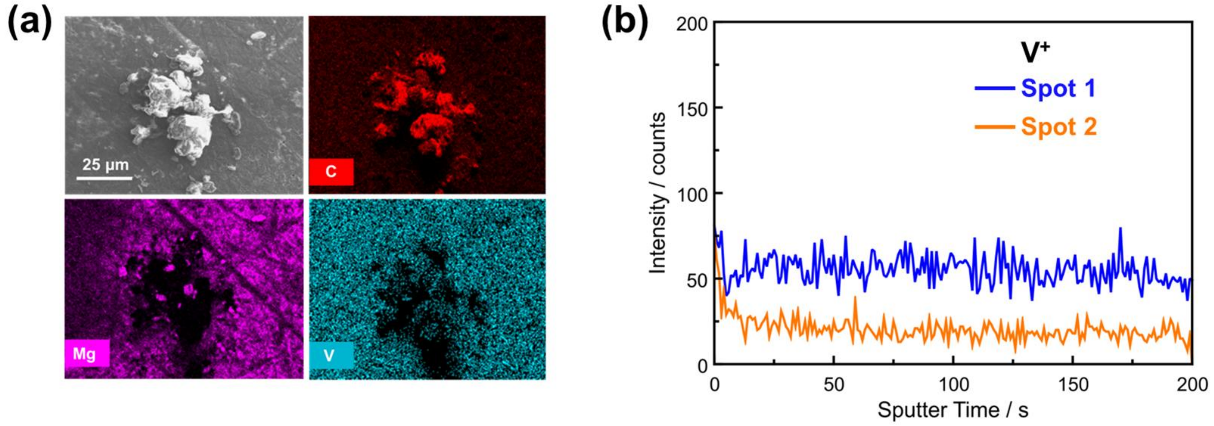

Figure S7. (a) SEM-EDX mapping of Mg metal surface after 1st cycle in three-electrode cell with VO_2 as the cathode. (b) ToF-SIMS analysis of V^+ spectra of Mg metal surface taken at two different spots, indicates the presence of vanadium.

Table S1. Elemental ratios estimated from the ICP-OES analysis on VO_2 cathode

Sample	Mass Ratio (%)		**Relative Atomic Ratio**	
	Mg	V	**Mg**	**V**
Pristine	<0.005	1.3	**0.00**	**1.00**
1st Discharge	0.019	1.3	**0.03**	**1.00**
1st Charge	<0.005	1.4	**0.00**	**1.00**
10th Discharge (0.3 V vs. Ag/Ag+)	0.079	1.2	**0.14**	**1.00**
10th Discharge (0.5 V vs. Ag/Ag+)	0.074	1.8	**0.09**	**1.00**

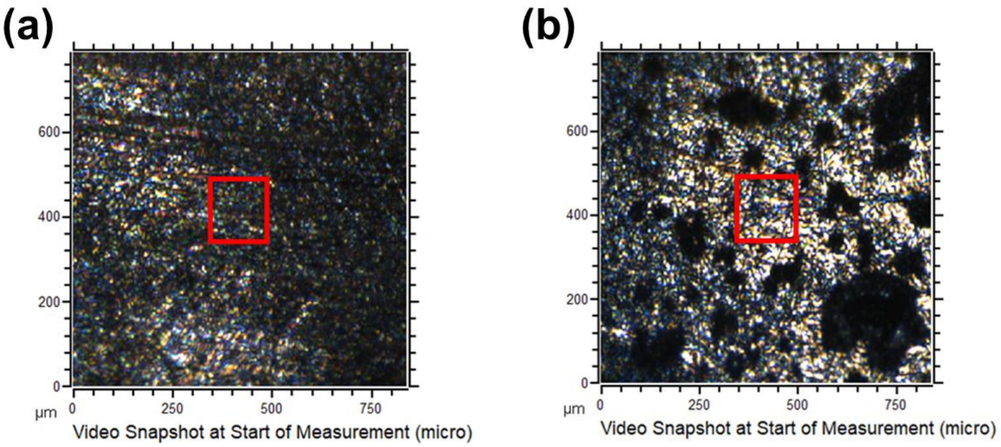

Figure S8. ToF-SIMS analysis area on Mg metal surface cycled with 0.3 M Mg[Al(hfip)$_4$]$_2$ in diglyme electrolyte in (a) symmetric cell and (b) in full cell with VO_2 as a cathode, respectively.

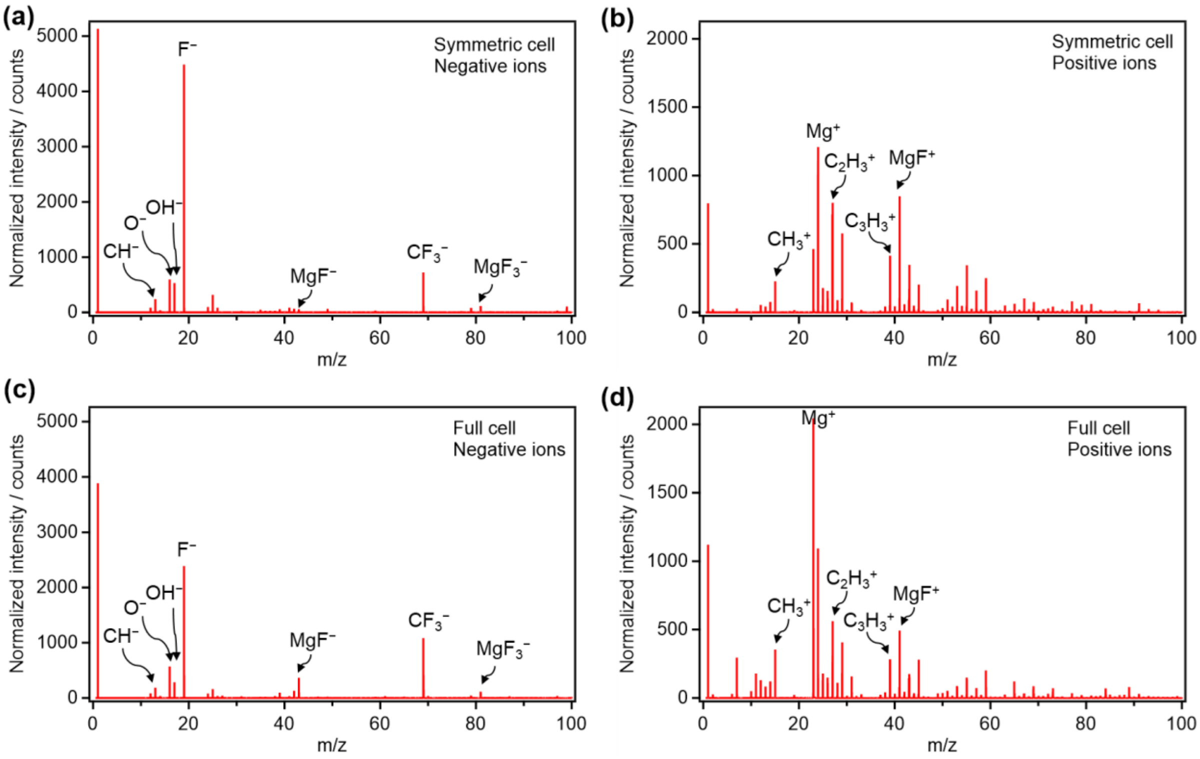

Figure S9. ToF-SIMS surface spectra of Mg metal cycled with 0.3 M Mg[Al(hfip)$_4$]$_2$ in diglyme electrolyte in (a), (b) symmetric cell and (c), (d) in full cell with VO_2 as a cathode, respectively.
